# A semantic segmentation model for road cracks combining channel-space convolution and frequency feature aggregation

**DOI:** 10.1038/s41598-024-66182-y

**Published:** 2024-07-11

**Authors:** Mingxing Zhang, Jian Xu

**Affiliations:** School of Electronics and information, Xi’an Polytechnic University, Xi’an, 710048 China

**Keywords:** Road crack detection, Deep learning, Frequency feature aggregation, Image segmentation, Neural networks, Computer science, Electrical and electronic engineering

## Abstract

In transportation, roads sometimes have cracks due to overloading and other reasons, which seriously affect driving safety, and it is crucial to identify and fill road cracks in time. Aiming at the defects of existing semantic segmentation models that have degraded the segmentation performance of road crack images and the standard convolution makes it challenging to capture the spatial and channel coupling relationship between pixels. It is difficult to differentiate crack pixels from background pixels in complex backgrounds; this paper proposes a semantic segmentation model for road cracks that combines channel-spatial convolution with the aggregation of frequency features. A new convolutional block is proposed to accurately identify cracked pixels by grouping spatial displacements and convolutional kernel weight dynamization while modeling pixel spatial relationships linked to channel features. To enhance the contrast of crack edges, a frequency domain feature aggregation module is proposed, which uses a simple windowing strategy to solve the problem of mismatch of frequency domain inputs and, at the same time, takes into account the effect of the frequency imaginary part on the features to model the deep frequency features effectively. Finally, a feature refinement module is designed to refine the semantic features to improve the segmentation accuracy. Many experiments have proved that the model proposed in this paper has better performance and more application potential than the current popular general model.

## Introduction

Cracks are common defects in highways, and their causes are more complicated; temperature changes, excessive loads, and subsidence of the soil base can all lead to cracks in highways. If the road surface is not sealed and repaired in time, rainwater and other debris will enter the surface structure and roadbed along the cracks, causing structural damage to the road and shortening the service life. In addition, more severe diseases caused by road cracks hide huge safety hazards and affect driving safety. Regular detection of road cracking is significant for extending road life and maintaining driving safety^1^.

Early road crack detection for manual observation, which is time-consuming and inefficient, must be improved to meet the increasing demand for road maintenance^2^. In recent years, computer vision-based image processing techniques have gradually replaced manual labor and become the mainstream road crack detection method^3^. Traditional image processing algorithms are based on machine vision, such as median filtering^4^, thresholding algorithm^5^, edge detection^6,7^, and so on. These algorithms are more sensitive to disturbing factors such as noise, lighting changes, and complex backgrounds in the image, and it is difficult to detect small cracks from complex images. It has become an inevitable trend to improve detection efficiency and accuracy, reduce the heavy workload of inspectors, and implement automatic pavement crack detection.

Subject to the shortcomings of traditional image processing algorithms, numerous road crack detection algorithms are based on semantic segmentation. Lin et al.^8^ proposed a practical segmentation framework for multi-scale crack feature learning named DeepCrackAT for the significant appearance variations and complex topology of cracks. The network uses hybrid null convolution to increase the sensory field, fuses multi-scale features, and introduces an attention mechanism to improve accuracy. However, it is not sensitive enough to the width variation of cracks. Benedetto et al.^9^ proposed a road crack segmentation algorithm based on Unet that focuses on the width variation of segmented cracks.

The above networks achieved good results in the road crack segmentation task but were relatively computationally complex. Qi et al.^10^ proposed GMDNet to solve the task of road crack segmentation in the face of discontinuities and irregularities. It introduces GhostNet as the backbone network, enhances the feature extraction capability by dynamic convolution, and keeps the computational cost low. Pang et al.^11^ proposed DcsNet, a real-time deep network for crack segmentation, which retains morphological information with scale invariance and constructs small-step shallow-detail branching to supplement the detailed information. Lightweight networks often sacrifice efficiency for efficiency, reducing the generalization ability.

In this paper, focusing on accurately segmenting road cracks in the complex context of concrete road grooves, tire marks, lane lines, etc., we propose a semantic segmentation model for road cracks that combines time-space convolution with frequency feature aggregation. First, a novel convolutional block is designed based on the residual idea for constructing the backbone network to perceive the crack feature information in gradual downsampling. Secondly, a frequency feature aggregation module is added in the depth of the network to aggregate the crack body and edge information, respectively, to enhance the network’s ability to recognize the crack pixels. Finally, to address the defects of feature redundancy and loss of critical features in multi-scale feature fusion, a multi-layer perceptron is first used to generate weight matrices to aggregate multi-dimensional features, and then an attention feature refinement module is designed to refine the multi-dimensional features to distinguish the background further. We evaluate the model proposed in this paper on the self-built dataset and open source dataset, respectively, and the experiments show that the model proposed in this paper can distinguish the background from the cracks more accurately, which provides a way of thinking for the automatic detection of cracks. The main contributions of this paper are as follows.

**(1)**A new convolutional block is designed for crack feature extraction that reduces convolution parameters with guaranteed performance.

**(2)**A frequency feature aggregation module is designed for crack characteristics. It uses a windowing method to solve the problem of input mismatch in the frequency domain while considering the complex frequency of features. Modeling the depth feature frequency by channel attention improves the defect that makes distinguishing between similar pixel points in the time domain difficult and enhances the crack edge features.

**(3)**An attention feature refinement module is proposed to reduce redundancy in multiscale feature fusion and reduce useless features from swamping valuable features. The crack features are refined to improve the segmentation accuracy.

## Related work

This chapter describes the related work to the design of deep learning-based road crack segmentation modeling tasks. For most of the work, the technical route includes data preprocessing, model optimization, and improving segmentation accuracy by incorporating practical application scenarios.

### Data preprocessing

Data preprocessing plays a crucial role in deep learning and directly affects the performance and effectiveness of the model. With the application of semantic segmentation models in road crack detection, researchers are often concerned about the quality of crack image acquisition. For example, the model’s performance drops dramatically under intense light or high noise. Moreover, road cracks have apparent geographical differences, and different construction materials and construction methods lead to variable road quality. Although there are currently many open source datasets, such as Crack500, Crack Tree, Crack Forest Dataset, etc., the quality of the datasets varies greatly. Therefore, it is not easy to guarantee the generalization performance of models trained on these datasets.

In order to ensure the segmentation performance of the model in practice,^12^ proposed a non-local filtering denoising method with fuzzy self-similarity weight estimation to remove various scattering noises on the dataset.^13^ used the non-local self-similarity of learnable depth features to train the regularization layer and reduce the distribution bias of the dataset effectively.^14^ proposed a twin transform network for image denoising to remove noise and artifacts from pictures. Although all the above networks have good denoising effects, they are not designed for crack images, and the denoising performance on the road crack dataset still needs to be further verified. In order to solve the above problems, random cropping, random rotation, random scaling, and other methods are usually used in engineering to enhance the data. However, these methods are too simple and cannot solve the problems of jitter, noise, and ghosting that occur during the acquisition of the road crack dataset well. Therefore, this paper uses intensity operations^15^ to reduce the deviation of the dataset’s luminance distribution and smooth the image’s noise while extending the dataset by combining the above methods. Data augmentation enhances the model’s training process, prevents the model from falling into local optimal solutions, and ensures the segmentation accuracy of the model. The formula for modifying the image brightness is as follows:

1 $$\begin{aligned} \begin{aligned} argminT\Vert {T-S}\Vert _2^2+\lambda \left( \Vert \nabla _xT/exp\left( \frac{(\nabla _x\Im _{\sigma 2}(T)^2)}{2\sigma _1^2}\right) \Vert \right. \\ \left. +\Vert \nabla _yT/exp\left( \frac{(\nabla _y\Im _{\sigma 2}(T)^2)}{2\sigma _1^2}\right) \Vert \right) \end{aligned} \end{aligned}$$

where *T* denotes the filtered image, and $\nabla _x,_yT$ is the differentiation of *T* in the *x*,*y* direction. The denominator is a Gaussian kernel of width $\sigma _1$. In the Gaussian kernel, before differentiation in the *x* or *y* direction, we also apply a Gaussian filter $\Im _{\sigma 2}{(\dot{)}}$ on *T*, where $\sigma _2$ is the spatial width. *S* is the original image, and $\lambda$ is the balance parameter. The first term is the L2 parametric fidelity term, which guarantees the overall similarity between the filtered and original images.

### Backbone networks

The backbone network plays a crucial role in neural networks^16^. It is responsible for extracting high-level features from the input data critical to the network’s final output. However, as the network depth increases, gradient vanishing occurs, causing the model performance to degrade drastically. The design of ResNet50^17^ has significant advantages in solving the gradient vanishing problem. The introduction of residual connectivity makes the network deeper and improves model performance and scalability. ResNet-50 performs well in several computer vision tasks, with excellent training results and many parameters.MobileNet V3^18^, a well-designed lightweight network model with streamlined parameters and fast computation speed, aims to deploy neural networks to mobile devices efficiently. Its innovation is the introduction of the lightweight SE(Squeeze and Excitation)^19^ attention mechanism, which enhances the expressive power of the network by explicitly modeling the interdependence of the convolutional feature channels. At the same time, the SE attention mechanism can strengthen the features that are beneficial to the current task and suppress the features that have less impact on the task.GhostNet^20^, on the other hand, focuses on solving the feature map redundancy problem in conventional convolution. Its core idea is to generate richer features with fewer parameters. The feature maps are first obtained through small-scale convolutional operations, and then linear transformations are applied to each layer to generate Ghost feature maps. Finally, the original and Ghost feature maps are superimposed on the channel to meet the demand for computational efficiency.

The backbone mentioned above networks have eye-opening advantages but have limited capability in crack feature extraction tasks. Therefore, designing a specialized feature extraction network for road crack segmentation tasks becomes essential. Based on this, this paper designs a channel-space convolution-based backbone network based on the residual idea of ResNet-50, which focuses on the spatial and channel features of the cracks and dramatically enhances the ability to recognize the crack pixels.

**Figure 1:**
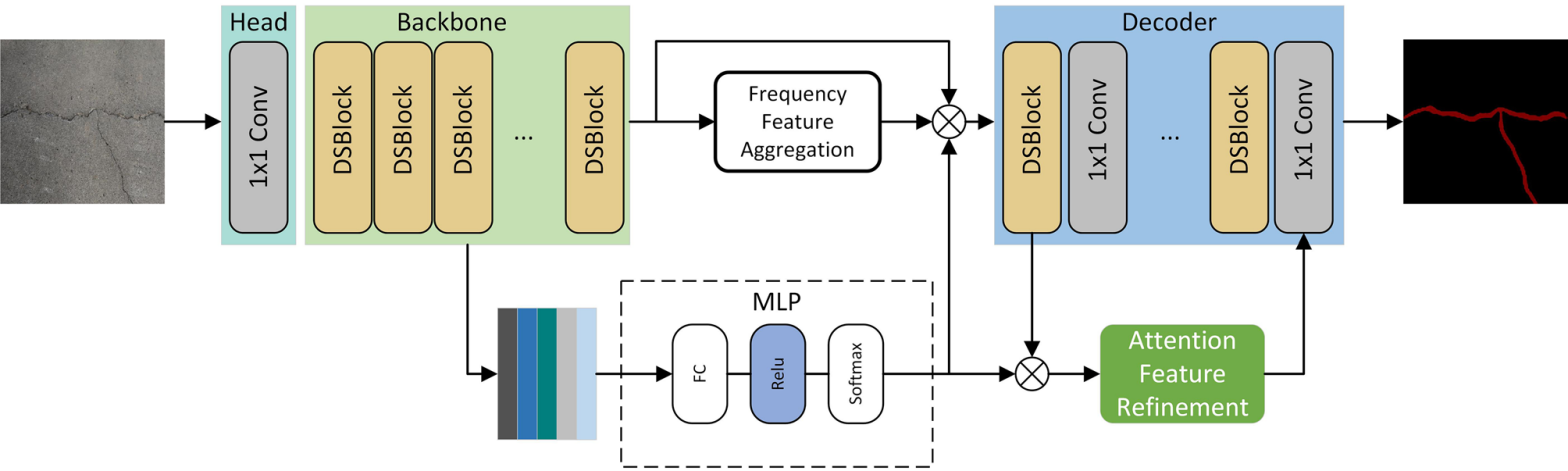
Overview of the model.

**Figure 2:**
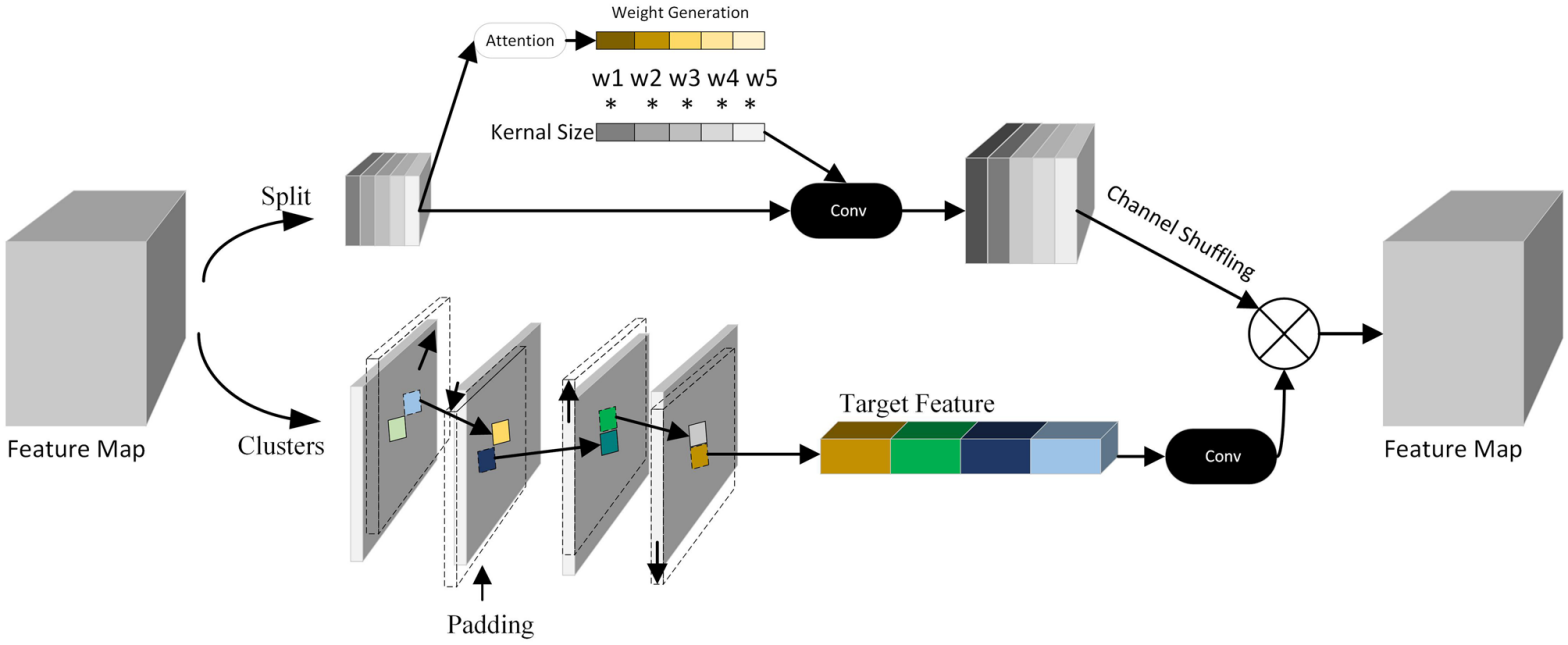
Channel-space convolution.

## Methodology

In this paper, a semantic segmentation model for road cracks combining channel-space convolution and frequency feature aggregation is proposed to accurately segment road cracks in the complex context of concrete roads with carved grooves, tire marks, and dirt. The model consists of an encoder, a frequency feature aggregation module, and a decoder, as shown in Fig. 1.

### Channel-space convolution

The traditional convolution operation has a limited sensory field, which can only consider the local region of the convolution kernel size and lacks the perception of the global information. In the depth feature map, the importance of different channels is different, and the convolution operation using the exact size of the convolution kernel increases many useless features and slows down the computation speed. At the same time, standard convolution is limited in the perception of structural connections in pixel space. Conventional convolutional operations perform a weighted summation of local pixels at each location but do not directly model the spatial structural connections between pixels. To solve the above problem, this paper proposes a channel-space convolution (CSConv), as shown in Fig. 2. CSConv is divided into two parallel channels, which process the channel features and spatial features, respectively. The upper channel first splits the feature map by channel, then uses attention to evaluate the importance of each channel to generate weights, and multiplies the weights with the convolution kernel so that the convolution kernel has a dynamic size. The feature map is then convolved at this point, which can discard unimportant features and enable the encoder to extract road crack features better. To achieve spatial feature aggregation, the lower channel introduces a parameter-free spatial displacement operation that aligns the feature map to the neighboring features along the channel direction. Firstly, the feature maps are grouped, and spatial displacements are applied to the feature channels of different groups to improve the aggregation of neighboring features at the corresponding positions of the convolution. Finally, the features extracted from the two channels are spliced to fuse the cracked channel-space features.

**Figure 3:**
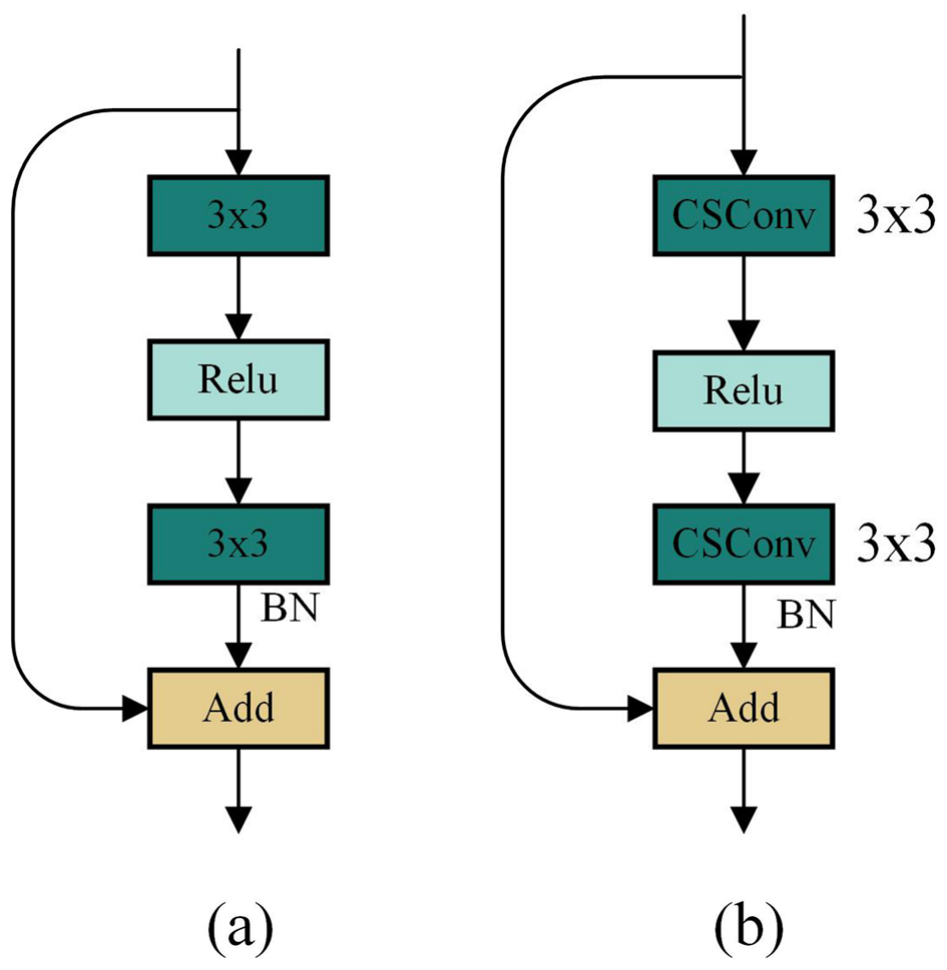
Improved residual convolution block. (**a**) Standard residual convolution block (**b**) Improved residual convolution block.

The residual convolution block is constructed using CSConv, which can better encode road crack features, as shown in Fig. 3. The improved residual block replaces the original two $3\times 3$ standard convolutions with CSConv to enhance the feature extraction capability. Based on the improved residual convolution block, we constructed the backbone network to extract the depth features of road cracks.

### Frequency feature aggregation module

**Figure 4:**
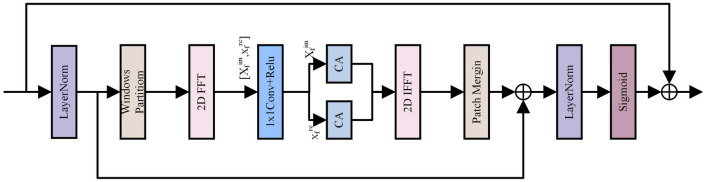
Frequency feature aggregation module.

In the time domain, the influence of the complex background can confuse the boundary between the crack and the background, making crack detection more difficult. Moreover, the time-domain signal mainly focuses on the signal variation in time. In contrast, the understanding of the structural information of the signal in space is limited, which makes the model inaccurate enough to detect delicate structures such as cracks. To address the above problems, this paper proposes the Frequency Feature Aggregation Module (FFAM), as shown in Fig. 4, which first standardizes the input feature layers and averages the input distribution. In order to overcome the feature loss due to frequency input mismatch, we first split the feature map uniformly into windows of size *N*, each of which is $S_N = [B, C, N, N]$. These windows are then fast Fourier transformed to the frequency domain:

2 $$\begin{aligned} \begin{aligned}{}&\ X_f(b,c,u,v)=F(S_N)&\\&\ =c(u)c(v)\sum _{h=0}^{N-1} \sum _{w=0}^{N-1} S_N{(b,c,h,w)}e^{-j2\pi (\frac{uh}{N}+\frac{vh}{N})} \end{aligned} \end{aligned}$$

where F(*) denotes the Fast Fourier Transform, *b* and *c* denote the batch and channel indices, and *u* and *v* denote the u-th horizontal and v-th vertical frequencies in the spectrum. In order to extract the depth features of the road cracks, we considered both the real and imaginary parts of the complex frequencies. We aggregated the frequency features using a convolution operation. The processing of any of the complex frequencies is as follows:

3 $$\begin{aligned} \begin{aligned} X_f1r=(X_fr\times K_r)-(X_fr\times K_i) \end{aligned} \end{aligned}$$

4 $$\begin{aligned} \begin{aligned} X_f1i=(X_fi\times K_i)-(X_fi\times K_r) \end{aligned} \end{aligned}$$

**Figure 5:**
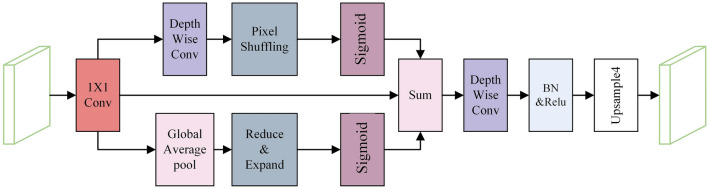
Attention feature refinement module.

where *X*1r and *X*1i denote the real and imaginary parts of the frequency, and *K*r and *K*i denote the real and imaginary parts of the complex convolution kernel. The road crack features in the spectrum are then modeled using the channel attention mechanism to highlight valuable features in the frequency channel. The windowed frequency feature maps are inverted two-dimensional fast Fourier transformed to merge the windows and restore the feature map size to [*B*, *C*, *H*, *W*]. Finally, the feature attention maps are generated using sigmoid, and the generated attention maps are multiplied with the original feature maps to enhance the crack features in the feature maps.

### Attention feature refinement module

Roadway cracks usually present different shapes, including linear, reticulated, and tortoise-fractured. Linear cracks are usually elongated cracks extending along the direction of the roadway. In contrast, mesh cracks present a network of intersecting cracks, and tortoise cracks present tiny cracks similar to tortoise cracks. As the network deepens, rich semantic features of cracks can be extracted from the backbone network, which is crucial for distinguishing cracks from the background. However, as the localized features of the cracks are extracted, the spatial dimension of the feature map keeps shrinking, leading to the loss of spatial correlation information.

Therefore, this paper introduces the Attention Feature Refinement Module (AFRM), as in Fig. 5, which aims to alleviate the semantic differences between the feature maps extracted by the backbone network, enhance their spatial relevance, and reduce redundant features. First, the feature maps generated by the backbone network are spliced, and a Multilayer Perceptron(MLP) augments the weights of the underlying useful features generated attention map, which is then multiplied with the augmented feature maps of the frequency features as input to the AFRM. The AFRM consists of spatial branches and channel branches. The spatial branch first generates the spatial feature maps of the cracks using deep convolution, then extracts the inter-pixel correlations by pixel blending operation and generates the attention maps by Sigmoid function. The channel branch first performs global average pooling of the feature maps, converted into a tensor of size $1 \times 1 \times C$ (where C is the number of channels). Subsequently, the number of channels is reduced to one-third of the original number of channels by reducing and expanding the layers, which are then recovered to enhance the semantic details of the channels, thus improving the segmentation of cracks. Finally, the feature maps are residually concatenated with the attention maps generated by the two branches to prevent network degradation, and convolutional layers and upsampling generate the final semantic segmentation map of road cracks.

### Ethics approval

The authors declare that this manuscript was not submitted to more than one journal for simultaneous consideration. The submitted work is original and not has been published elsewhere in any form or language.

### Consent to participate

The authors declare that they participated in his paper willingly.

## Experiment and analysis

### Data sets and experimental platforms

**Figure 6:**
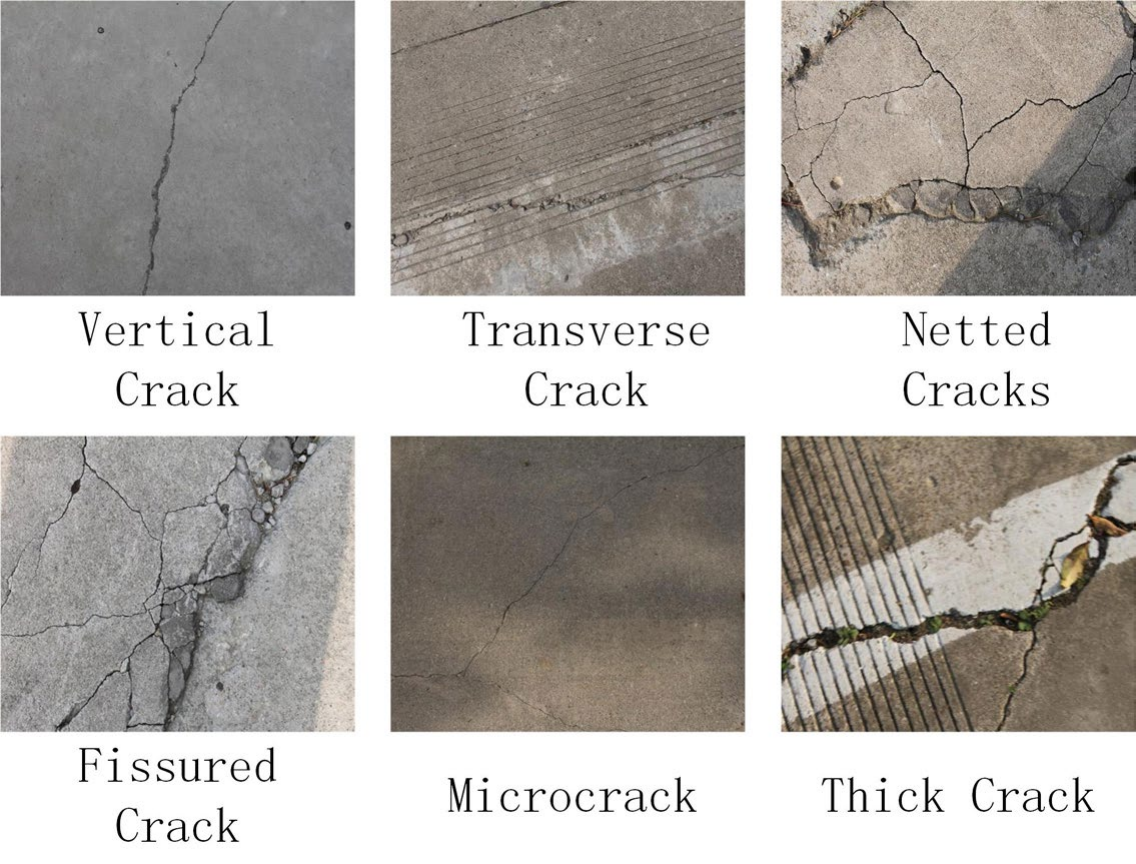
Self-constructed dataset.

In order to validate the effectiveness of the network model proposed in this paper, we introduced a pavement crack dataset. This dataset was captured by a German company’s industrial face array camera with pixel resolution $512 \times 512$. In order to improve the robustness of the network model, we selected different lighting conditions, shadows, clutter, and other scenes for shooting the pictures, and a total of 2026 pavement crack pictures were collected. To improve the model’s generalization ability, we use data enhancement methods of random cropping, rotation, scaling, and injecting Gaussian noise to extend the dataset. Intensity operation methods were used to improve the dataset’s quality and expand it, resulting in a total of 6400 crack images with a resolution of $256 \times 256$. The pavement crack dataset is divided into a training set and a test set in the ratio of 8:2, and Labelme software is used for labeling. Some of the crack images are shown in Fig. 6.

The programming environment for the experiments is Python 3.6 and Torch version 1.10.8. The hardware environment is an Intel I7-7800X CPU equipped with an NVIDIA GeForce RTX3090 graphics card with 24GB of video memory and 16GB of RAM. The training is optimized using the Adam optimizer to prevent the model from falling into the local optimal point during the training process.

### Segmentation assessment criteria

This study employs classical evaluation metrics commonly utilized in semantic segmentation to assess segmentation models with varying depths. These metrics encompass the Mean Intersection over Union (mIoU), mean Pixel Accuracy (mPA), and Precision. These metrics are quantified using the following equation:

5 $$\begin{aligned}&\ mIoU=\frac{1}{2}\sum _{i=0}^1\frac{TP}{TP+FP+FN}&\end{aligned}$$

6 $$\begin{aligned}&\ Precision=\frac{TP}{TP+FP}&\end{aligned}$$

7 $$\begin{aligned}&\ mPA=\dfrac{1}{n}\sum _{i=1}^{n}APi&\end{aligned}$$

where *TP* represents the count of true positives, *FP* denotes the count of false positives, and *FN* denotes the count of false negatives. It stands for the pixel accuracy of each image, which is aggregated and averaged to yield the average pixel accuracy across the entire dataset. *Pi* represents the accuracy of the identified pixel points corresponding to pavement cracks in the image, determined by comparing them with the pixels in the ground truth labels. Additionally, this paper introduces Floating Point Operations Counts(FLOPs) and Params to gauge the computational complexity of the network.

###  Model performance evaluation

In this section, the proposed road crack semantic segmentation model is validated. It is compared with the current popular semantic segmentation models Unet^21^ , ResUnet^22^ , DeepLabV3^23^ , and AttentionUnet^24^ , and the experimental results are shown in Table 1. Figure 6 shows the performance of each model on the self-built dataset, Crack Tree(CT)^25^ and Crack Forest Dataset(CFD)^26^.

**Table 1:** Comparison of training results of different models on each dataset.

Database	Module	mIoU (%)	mAP (%)	Precision (%)	Params (M)	FLOPs (G)
Self-built	Unet	77.83	86.63	74.42	24.891	46.025
	ResUnet	78.37	86.36	75.59	43.933	54.459
	DeepLabV3+	77.51	90.56	82.44	5.813	6.608
	AttnUet	80.72	87.54	87.11	57.16	135.34
	Ours	80.93	90.88	85.99	12.836	6.846
CFD	Unet	66.53	76.85	75.75	24.891	46.025
	ResUnet	67.03	75.56	77.01	43.933	54.459
	DeepLabV3+	63.28	72.80	73.40	5.813	6.608
	AttnUet	65.11	76.70	74.12	57.16	135.34
	Ours	69.57	81.87	77.15	12.836	6.846
CT	Unet	66.75	71.13	82.53	24.891	46.025
	ResUnet	66.67	70.76	81.85	43.933	54.459
	DeepLabV3+	64.10	69.05	77.93	5.813	6.608
	AttnUet	66.57	70.41	83.69	57.16	135.34
	Ours	67.09	71.39	83.07	12.836	6.846

As can be seen from Table 1, the model proposed in this paper outperforms the other models on each dataset, achieving a balance between performance and parameters. Regarding the number of parameters and FLOPs, the model proposed in this paper has only 12.836M parameters and 6.846G FLOPs, which indicates that the model can characterize more cracks with relatively few parameters. Although DeepLabV3+ has a smaller number of parameters, it is not as good as the model proposed in this paper in terms of segmentation performance. In terms of segmentation accuracy, the accuracy of the model proposed in this paper is 85.99%, 77.15%, and 83.07% on the three datasets, respectively. Compared with Unet and ResUnet, the model proposed in this paper significantly improved, leading by 11.57% and 10.4% on the self-built dataset, respectively. Compared with DeepLabV3+, the atrous convolution dramatically reduces the number of parameters and the segmentation performance.AttentionUnet adds multiple attention mechanisms to Unet, resulting in the highest accuracy, but also increases the computational effort of the model, which is challenging to train. In contrast, the model proposed in this paper is much less complicated to train while not sacrificing too much accuracy.

In a comprehensive comparison, the model proposed in this paper is ahead of the current popular models in terms of performance. This is because the proposed model is constructed by the lightweight CSConv, which significantly reduces the parameters and computation of the model while maintaining good feature extraction capability. The added frequency feature aggregation and attention feature refinement modules further improve the model segmentation accuracy.

**Figure 7:**
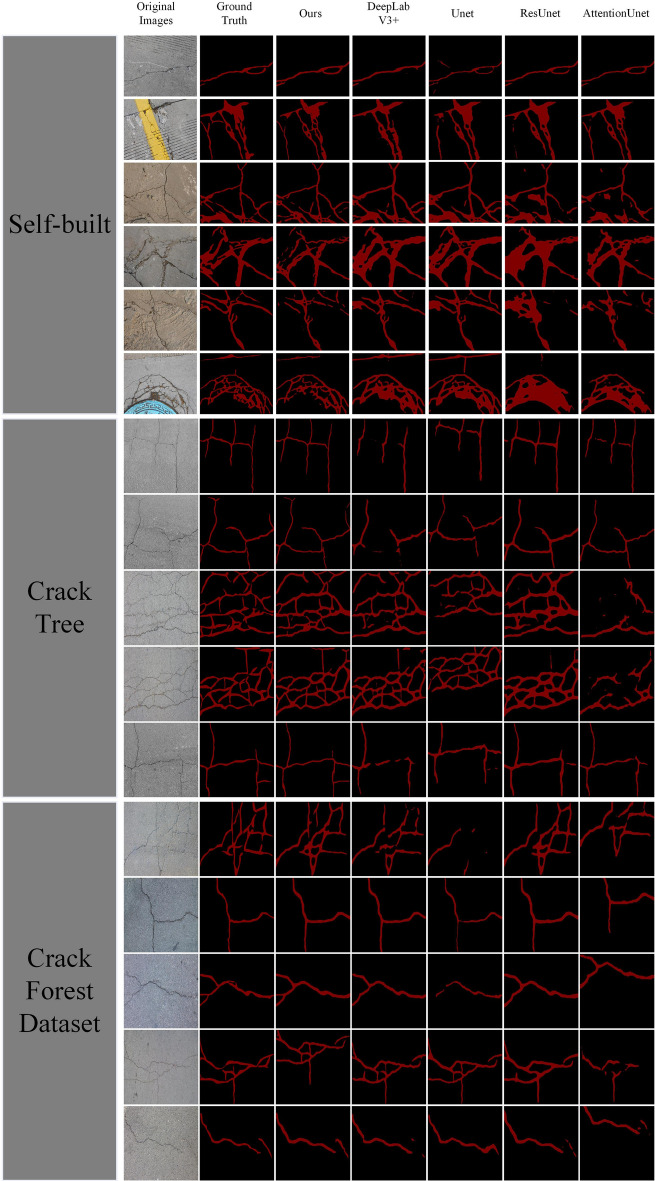
Visualization of model segmentation results.

Figure 7 shows the visualization results of each model segmentation. Column 1 is the original image, column 2 is the image annotation, and columns 3 to 5 are our model, DeepLabV3+ model, Unet model, ResUnet model, and AttentionUnet model, respectively. Five typical images are extracted from each dataset, and comparing the image segmentation results, the model in this paper has a more robust generalization performance and is more accurate for segmenting pavement cracks with complex backgrounds.DeepLabV3+ has a more severe under-segmentation when facing complex cracks, especially at the interface of cracks; this is due to the fact that DeeplabV3+ does not consider the correlation between the spatial information and the perception field and cannot take full advantage of the correlation between the shallow field and the spatial information, and cannot take full advantage of the shallow field. Unet and ResUnet can only obtain the local context information by standard convolution, and the global features of the cracks are not well preserved.AttentionUnet uses the multiple attention mechanism to model the spatial and channel contexts, and it can obtain good segmentation, but under-segmentation is serious when dealing with complex cracks. In summary, the model in this paper has better segmentation performance than other models for both simple and complex cracks.

### Backbone network performance comparison

**Table 2:** Backbone network performance comparison.

Backbone		mIoU (%)	mAP (%)	Precision (%)	Params (M)	FLOPs (G)
ShuffleNetV2		78.52	88.72	84.50	10.25	6.375
MobileNetV3		77.66	88.79	83.76	5.939	2.202
EfficientNetV2		76.09	84.51	85.09	24.351	8.418
GhostNetv2		78.40	88.72	84.79	19.722	3.553
MobileViT		78.80	89.12	85.02	8.892	6.150
Ours		80.93	90.88	85.99	12.836	6.846

**Table 3:** Model ablation experiments.

		mIoU (%)	mAP (%)	Precision (%)	Params (M)	FLOPs (G)
ResNet50		74.52	80.72	81.50	43.933	23.012
CSConv		75.52	90.02	82.54	7.461	5.875
+FFAM		79.47	83.33	83.21	8.408	5.301
+AFRM		78.98	89.51	84.87	10.513	6.312
Ours		80.93	90.88	85.99	12.836	6.846

This section evaluates the proposed model’s feature extraction capability compared to the popular backbone networks. These include ShuffleNetV2^27^,MobileNetV3^28^, GhostNet^29^, and EfficientNetV2^30^, which are backbone networks based on convolutional neural network(CNN) architectures, and MobileViT^31^, which is a backbone network that mixes CNN and Vision Transformer. Each model is trained on the self-constructed crack dataset, and the experimental results are shown in Table 2.

According to Table 2, the models proposed in this paper are superior in crack feature extraction capability compared to other popular backbone networks. This is due to CSConv’s modeling of local features of road cracks, which enhances the connection between feature channels and the spatial connection between crack pixels. The convolutional block proposed in this paper is based on CNN architecture, which compensates for the lack of standard convolution in local spatial feature extraction by spatial displacement operation compared to other CNN-based backbone networks, making the backbone network more sensitive to the spatial features of road cracks, and capable of extracting the detailed features of the cracks more finely. Compared with MobileViT, the FLOPs of our model increase by only 0.696 G but improve the mIoU by 2.13%.In contrast, the fixed-size convolutional kernel cannot efficiently capture features of different scales and sizes and has a limited receptive field. In contrast, the dynamic convolutional kernel of CSConv can pay targeted attention to the crack features, which improves the segmentation accuracy.

### Grad-CAM visualization results

**Figure 8:**
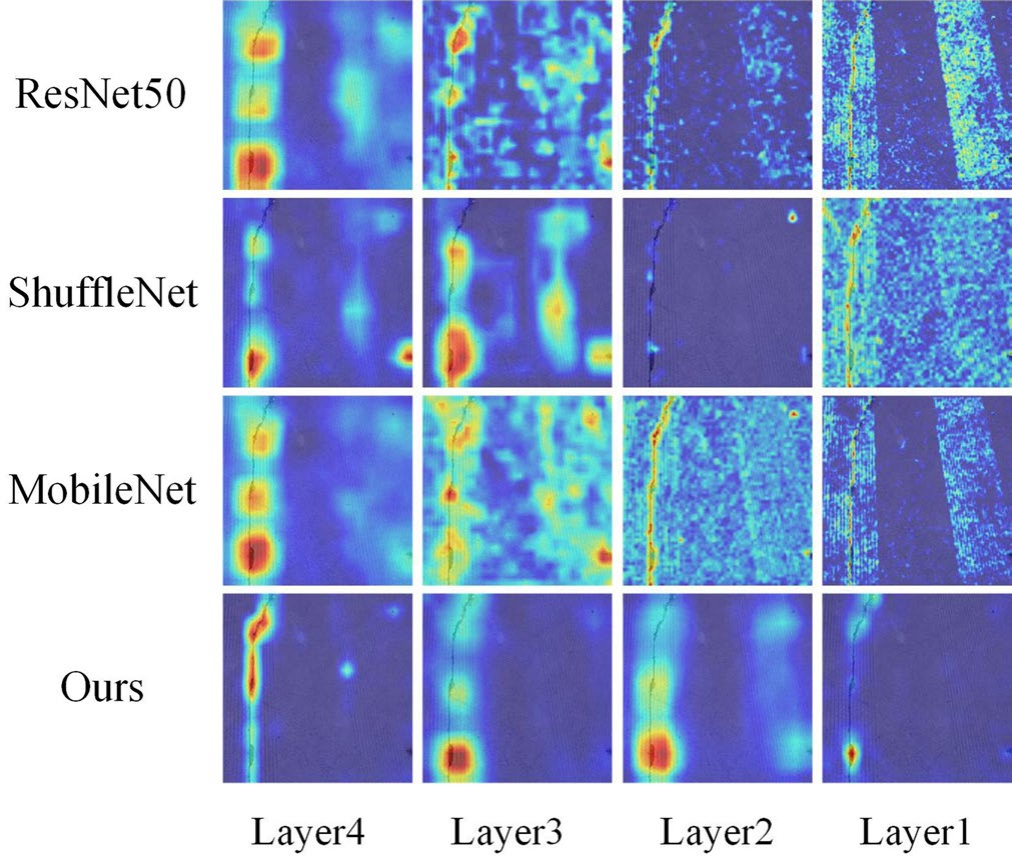
Visualization of model segmentation results.

In order to analyze the regional weights of road crack images, as well as to compare the changes in the weights of crack images from different models, this paper introduces gradient-weighted class activation mapping (Grad-CAM) to generate a saliency mapping for each convolutional layer, thus highlighting the critical regions that influence the prediction of crack images. As shown in Fig. 8, ResNet50, ShuffleNet, MobileNet, and ours are selected for heat map visualization comparison, showing each layer’s crack heat map after feature extraction, respectively.

As seen in Fig. 8, these four models differ in their attention to cracks and their handling of the effect of carved grooves. Specifically, ResNet50 pays more attention to cracks but handles the effect of incised slots poorly, mistaking incised slots for cracks and leading to errors.ShuffleNet overcomes the effect of incised slots but pays insufficient attention to cracks and suffers from defects in crack feature recognition in the global region. The MobileNet model focuses on the global region but has difficulty distinguishing between incised slots and cracks. The crack feature part will be lost with increased network depth, resulting in a poor segmentation effect. In contrast, the model proposed in this paper focuses more on the crack region and ignores the invalid region, reflecting the powerful feature extraction capability of CSConv. CSConv can effectively fuse the cracks’ spatial and channel features and enhance the valid region’s features, thus improving the segmentation capability.

### Ablation experiments

In order to verify the effect of different modules on segmentation ability, ResNet50 is used as the baseline, to which CSConv, frequency feature aggregation module, and attention feature refinement module are added step by step and trained on a self-constructed dataset. The experimental results are shown in Table 3. CSConv improves the shortcomings of standard convolutional spatial modeling and mainly contributes to the average pixel accuracy of the model. The spatial shift operation achieves the aggregation of nearest-neighbor features at the corresponding position, which improves the average pixel accuracy of the model by 9.3% and does not add many parameters because the shift operation is parameterless.FFAM enhances the edge contrast of the cracks in the frequency domain, and windowing patches enhance the features’ local details, contributing a 4.95% mean intersection over union to the model.AFRM reduces semantic gaps between underlying feature maps, which makes the model pay more attention to the details of the road cracks and contributes 3.37% Precision to the model. Overall, the backbone network of the model in this paper accounts for only 29.2% of the number of parameters and 29.7% of the FLOPs of ResNet50. However, each module improves the model’s segmentation capability.

## Conclusion

In this paper, we pinpoint the limited performance of existing semantic segmentation models in road crack detection tasks, and propose a semantic segmentation model for road cracks that combines channel-space convolution with frequency feature aggregation. By introducing a new convolutional block, the pixel-space relationship can be modeled more effectively in connection with the channel features to identify the crack pixels accurately. In addition, in order to enhance the contrast of crack edges, a frequency-domain feature aggregation module is proposed, which effectively solves the problem of mismatch of frequency-domain inputs and efficiently models the deeper frequency features by taking into account the effect of the imaginary part of the frequency on the features. Finally, a feature refinement module is designed to refine the semantic features to improve the segmentation accuracy further. The experimental results show that the proposed model outperforms the current popular generalized models for the road crack segmentation task and has superior performance and application potential.

## Discussion

We use a number of comparative experiments in the experimental section to demonstrate that the proposed model is fast and accurate for road crack detection, but we still want the proposed model to be robust in the face of more complex road cracks. Therefore, in this chapter, we examine the robustness of the proposed model under bad weather and other modal data. Table 4 records the training results of the model, and it can be seen that the model performance decreases dramatically under infrared spectral data, and it still cannot accurately detect simple cracks. The model’s robustness in rainy-day pavement crack detection is slightly better than that of infrared spectral data, but the segmentation accuracy is not enough.

**Table 4:** Validation of model performance on Infrared Spectrum and Rainy weather data.

	Dataset	mIoU	mAP	Precision
	Infrared spectrum	0.5373	0.5180	0.5504
	Rainy weather	0.6329	0.6679	0.7183

Exploring the reasons for the performance degradation, we suggest that, first, there is no standardized dataset of other spectral or severe weather road cracks in existence. The boundaries of road cracks in these cases have low contrast with normal pavements and significant noise effects. Second, the proposed model is not yet capable of handling extreme and complex road surface conditions such as infrared spectra or rainy weather. In future work, we will improve the model to address these shortcomings and strive to be able to achieve accurate detection of road cracks in all-weather conditions.

## Data Availability

The authors are not able to disclose the data at this time because the foundation program is still confidential. If any scholars would like to obtain data from this study, please contact the corresponding author. Email:zmx18792354440@163.com.

## Code availability

The project code cannot be made public for the time being.
